# Aggregation and Prion-Inducing Properties of the G-Protein Gamma Subunit Ste18 are Regulated by Membrane Association

**DOI:** 10.3390/ijms21145038

**Published:** 2020-07-16

**Authors:** Tatiana A. Chernova, Zhen Yang, Tatiana S. Karpova, John R. Shanks, Natalia Shcherbik, Keith D. Wilkinson, Yury O. Chernoff

**Affiliations:** 1 Department of Biochemistry, Emory University School of Medicine, Atlanta, GA 30322, USA; zenyang@mit.edu (Z.Y.); jshanks@emory.edu (J.R.S.); genekdw@emory.edu (K.D.W.); 2 Center for Cancer Research Core Fluorescence Imaging Facility, Laboratory of Receptor Biology and Gene Expression, National Cancer Institute, National Institutes of Health, Bethesda, MD 20892, USA; karpovat@mail.nih.gov; 3 Department of Cell Biology, Rowan University, School of Osteopathic Medicine, Stratford, NJ 08084, USA; shcherna@rowan.edu; 4 School of Biological Sciences, Georgia Institute of Technology, Atlanta, GA 30332-2000, USA; 5 Laboratory of Amyloid Biology, St. Petersburg State University, 199034 St. Petersburg, Russia

**Keywords:** prion, amyloid, mnemon, mating, G-protein, Sup35, Ste18, ubiquitin, phosphorylation, yeast

## Abstract

Yeast prions and mnemons are respectively transmissible and non-transmissible self-perpetuating protein assemblies, frequently based on cross-β ordered detergent-resistant aggregates (amyloids). Prions cause devastating diseases in mammals and control heritable traits in yeast. It was shown that the de novo formation of the prion form [*PSI*^+^] of yeast release factor Sup35 is facilitated by aggregates of other proteins. Here we explore the mechanism of the promotion of [*PSI*^+^] formation by Ste18, an evolutionarily conserved gamma subunit of a G-protein coupled receptor, a key player in responses to extracellular stimuli. Ste18 forms detergent-resistant aggregates, some of which are colocalized with de novo generated Sup35 aggregates. Membrane association of Ste18 is required for both Ste18 aggregation and [*PSI*^+^] induction, while functional interactions involved in signal transduction are not essential for these processes. This emphasizes the significance of a specific location for the nucleation of protein aggregation. In contrast to typical prions, Ste18 aggregates do not show a pattern of heritability. Our finding that Ste18 levels are regulated by the ubiquitin-proteasome system, in conjunction with the previously reported increase in Ste18 levels upon the exposure to mating pheromone, suggests that the concentration-dependent Ste18 aggregation may mediate a mnemon-like response to physiological stimuli.

## 1. Introduction

Amyloids are fibrous protein aggregates that cause devastating diseases in mammals. Prions are self-perpetuating transmissible (heritable or infectious) protein isoforms, in most cases based on amyloids [1,2]. In yeast and fungi, prions control heritable traits. Some yeast prions are clearly deleterious to the host [2,3], while other have been linked to potentially adaptive roles [4,5,6,7,8]. Supposedly, formation of heritable aggregates increases phenotypic diversity by altering a range of cellular processes that may have various phenotypic consequences. Non-heritable elements with the molecular basis that is similar to prions have been also identified. Some of them are responsible for the cellular memory and therefore termed “mnemons” [9]. Phenotypic switches controlled by prions or mnemons may play an important role when an organism faces an environmental stress or drastic physiological changes [10,11]. Formation and propagation of yeast prions are controlled by stress-related chaperones, protein quality control deposits, degradation pathways and cytoskeletal networks [12,13]. Environmental stresses initiate formation or loss of prions, possibly via affecting intracellular concentrations of prion-inducing proteins or auxiliary chaperones, and/or by promoting the assembly of misfolded prionogenic proteins at prion induction sites via heterologous secondary helpers [14,15]. 

In most cases, it remains unclear how prions or other prion-like assemblies arise in vivo. Yeast prion proteins commonly possess prion domains (PrDs) that are involved in prion propagation and are, in most cases, rich in N and/or Q residues [16]. It is likely that the multimerization of a misfolded prion-forming protein facilitates its conversion into an initial prion ‘‘seed.’’ Initial prion nucleation could be, therefore, promoted when the prion-forming protein is present at a high local concentration. Indeed, de novo formation of yeast prions is induced by transient overproduction of a prion-forming protein or its PrD (for reviews, see [10,13]). De novo prion nucleation could also be significantly enhanced by the presence of other QN-rich prions, or by simultaneous overproduction of other yeast proteins with QN-rich domains [17,18]. It is thought that pre-existing prion aggregates or aggregates of overproduced heterologous proteins represent initial nucleation centers for Sup35 aggregation [17,19] and/or sequester cofactors that usually antagonize prion formation by Sup35 [18,20]. Interactions between PrDs or PrD-like regions of various proteins, e.g., Pub1/TIA and Sup35 [21], as well as promotion of the aggregation of heterologous polyglutamines by endogenous yeast prions [22,23,24] indicate that various QN-rich proteins can be accumulated at particular cellular locations that turn into prion nucleation sites. Interactions between various amyloidogenic proteins have also been reported in mammalian systems, and some human amyloidoses (including Alzheimer’s disease) involve formation of amyloids by more than one protein [25,26]. 

One possibility is that some of heterologous prion inducers may act by targeting prionogenic proteins to certain intracellular locations. These locations may serve as prion nucleation sites where assembly of protein aggregates and their conversion into a prion form are facilitated. The actin cytoskeleton performs a central role in the trafficking and deposition of damaged and aggregated yeast proteins [27]. The yeast prion-forming protein Sup35 is implicated in the regulatory network involving the actin assembly factor Las17 [28] and was demonstrated to interact with several members of the cortical actin cytoskeleton [29,30]. Moreover, these interactions are critical for prion induction by excess Sup35. In an agreement with these data, we have shown that the ability of actin associated protein Lsb2 (Pin3) to aggregate and promote [*PSI*^+^] formation depends on its association with actin patches [31].

Another yeast protein shown to promote initial nucleation of the Sup35 prion when overexpressed [17] is Ste18, a γ-subunit (Gγ) of a G protein receptor, a highly conserved component of the eukaryotic signaling machinery, that plays a key role in a variety of cellular processes [32,33]. In yeast, Ste18 is involved in the pheromone-signaling pathway crucial for the process of mating [34]. Occupancy of the G-protein-coupled receptor (GPCR) Ste2 by peptide pheromone α-factor initiates signaling via release of a stimulatory Gβγ complex (Ste4-Ste18) from its inhibitory Gα subunit (Gpa1) [35]. Then, this Ste4-Ste18 dimer nucleates the formation of a protein complex composed of Ste5, kinases and other proteins important for cell polarization at the plasma membrane [34,35,36]. The formed complex triggers activation of the protein kinase cascade, resulting in the arrest of the cell cycle at G1 phase and redistribution of polarized cell growth in the direction towards the pheromone source, that is, a cell of the opposite mating type [34]. Thus, timely expression and proper localization of Ste18 is crucial for cellular mating. Normally, the abundance of Ste18 in the cell is low, however it is increased during prolonged exposure to pheromone [37], suggesting that effects of Ste18 overproduction could mimic some processes occurring at physiological conditions. Ste18 undergoes several posttranslational modifications (PTMs), such as: phosphorylation, modulating cellular levels of Ste18 [37,38]; lipidation, involved in the attachment of Ste18 to a membrane [39,40]; and ubiquitination [41]. While ubiquitination frequently regulates protein turnover, impact of ubiquitination on Ste18 levels has not been reported previously.

Here, we demonstrate that yeast Gγ (Ste18) protein can form detergent-resistant aggregates, potentially serving as “seeds” for the assembly of another misfolded amyloidogenic protein and promotion of its conversion into a prion. Moreover, aggregation and the prion-inducing properties of Ste18 are strictly dependent on its association with a membrane. In contrast to other aggregated proteins capable of cross-seeding amyloids, Ste18 aggregates failed to demonstrate heritability in our experimental system, thus resembling mnemons rather than prions. We also show that cellular levels of Ste18 are controlled by its ubiquitination via promoting degradation through Ubiquitin-Proteasome System (UPS). These data uncover the ability of a signaling protein to form assemblies with prion-inducing properties, and further emphasize the role of a specific intracellular localization in de novo nucleation of self-perpetuating protein aggregates. As components of the G-protein coupled signaling machinery, including Gγ, are conserved in a variety of eukaryotic organisms (including humans), these results could be applicable beyond yeast.

## 2. Results

### 2.1. Overproduced Ste18 Promotes de novo [PSI^+^] Induction and Forms Aggregates that Are Transiently Associated with Aggregated Sup35

**Figure 1:**
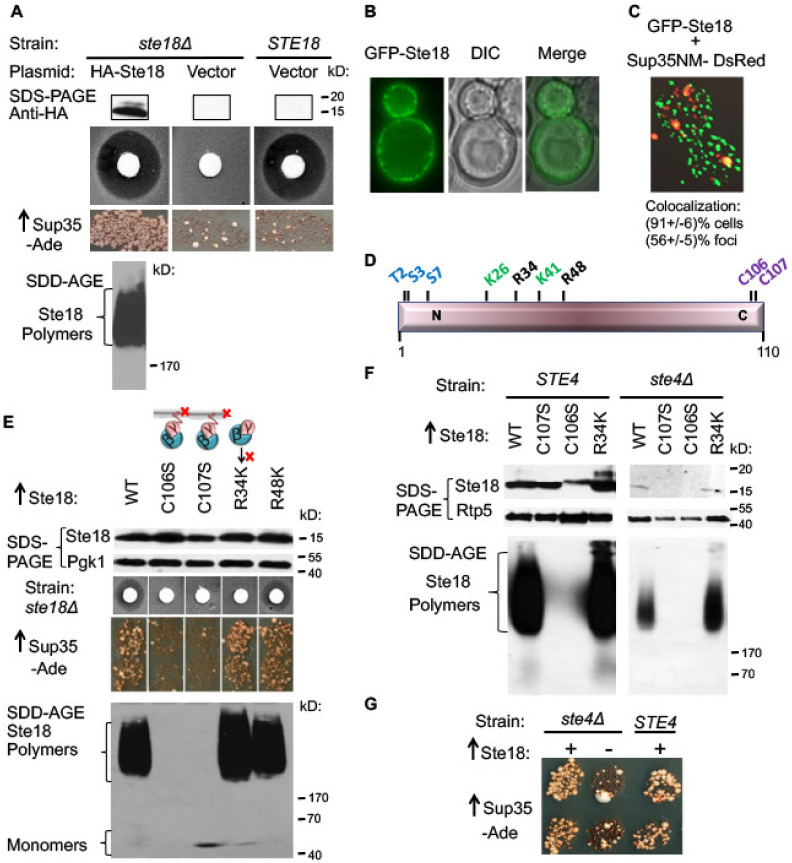
Ste18 induces [*PSI*^+^] prion formation and forms detergent resistant aggregates. (**A**) HA-Ste18 protein was expressed in Ste18 depletion strain (*ste18Δ*) and detected by Western blot with anti-HA antibody. Additional HA-positive band that migrated above the major band represents phosphorylated form of Ste18. Functionality of HA-Ste18 was analyzed by halo assay. A filter disc with α-factor solution was placed on the lawn as indicated. The zone of growth inhibition observed around the disc indicates the arrest of cell division after exposure to α-factor. Ste18 induces [*PSI*^+^] when overexpressed along with Sup35N as detected by growth on the medium without Ade (−Ade) following transient overproduction of both proteins. Rare Ade^+^ colonies appearing with the empty vector control represent background revertants. Overexpressed Ste18 forms detergent resistant polymers identified by SDD-AGE gel and Western blotting with anti-HA. (**B**) GFP-Ste18 forms punctate structures at the membrane periphery. Live cells with GFP-Ste18 were imaged with a 100× oil immersion objective on the Olympus IX81 microscope. Representative images are shown. (**C**) Some Sup35 aggregates are co-localize with Ste18 aggregates. Live cells with GFP-Ste18 and Sup35NM-dsRed were imaged on Delta Vision Core system; % of colocalization with a standardized error is shown. See also Supplementary Movie S1. (**D**) Structural organization of the Ste18 protein. Numbers correspond to amino acid positions. Post-translational modifications: T2, S3, S7-phophorylation; K26, K41-ubiquitination; C106-palmitoylation, C107-farnesylation. C106 and C107 are required for the binding of Ste18 to a membrane. R34, R48 are essential for signaling between Ste18/Ste4 complex and elements downstream of the pathway. (**E**) Effects of mutations on Ste18-dependent phenotypes. The *ste18Δ* strain was transformed with plasmids expressing either wild-type or mutant derivatives of Ste18 from *P_CUP1_* promoter. Cartoon illustrates the impact of mutations on membrane binding and downstream signaling. Levels of protein expression were compared by Sodium Dodecyl Sulfate - Polyacrylamide Gel Electrophoresis (SDS-PAGE) and Western blotting with anti-HA antibody. Pheromone sensitivity, [*PSI*^+^] induction and polymer formation in SDD-AGE were assessed as on panel. Mutants deficient in membrane binding cannot form detergent-resistant polymers and promote [*PSI*^+^] induction. Note that the correspondence of the molecular weights of monomers to markers is imprecise on SDD-AGE gels due to high diffusion. (**F**) Ste4 does not influence formation of the Ste18 polymers. Wild-type or mutant HA-Ste18 were expressed in either wild-type strain (*STE4*) or strain lacking Ste4 (*ste4Δ*). Protein levels were analyzed by SDS-PAGE, and formation of detergent resistant polymers was analyzed by SDD-AGE. Anti-HA antibody were used to detect HA-Ste18. Pgk1 was used as a loading control. (**G**) Ste4 does not influence [*PSI*^+^] nucleation by Ste18. Sup35N was overexpressed in wild-type strain (*STE4*) or strain lacking Ste4 (*ste4Δ*), either together with Ste18 (+) or without Ste18 (−). Formation of Sup35 prion form was detected as on panel (**A**).

As a γ-subunit of a G protein receptor, yeast Ste18 plays a key role in a in a pheromone-signaling pathway crucial for the process of mating [32,33]. Our experimental model employs the yeast strain of the mating type **a**, lacking the *STE18* gene on the chromosome and bearing the plasmid expressing the HA-tagged derivative of *STE18* from the copper-inducible promoter (*P_CUP1_*). We have confirmed that the HA-tagged Ste18 remains functional in signaling, as seen from its ability to control response to the mating pheromone of cells of the opposite mating type, α-factor. When a filter disc with α-factor solution is placed on the lawn of *ste18Δ* cells, expressing HA-Ste18, the zone of growth inhibition can be detected around the disc, indicative of the arrest of cell division after exposure to α-factor (Figure 1A). Previously, it was shown that that overexpression of Ste18 promotes formation of the prion state [*PSI*^+^] by yeast translation termination factor Sup35 (eRF3) in cells overproducing Sup35 or its derivatives containing the PrD (Sup35N) region, and lacking any other pre-existing prions such as [*RNQ*^+^] or [*PIN*^+^] [17]. Confirming these observations, we have demonstrated here (Figure 1A) that efficient [*PSI*^+^] induction is detected after simultaneous transient co-overproduction of Ste18 and Sup35 proteins in the [*psi^−^ pin*^−^] strain containing a reporter with a premature stop codon (UGA) in the *ADE1* gene, *ade1-14*, see [42]. In this system, growth on −Ade medium occurs due to partial loss of Sup35 function when a Sup35 prion forms, allowing the readthrough of a stop codon. The colonies growing on−Ade medium (Ade^+^ colonies) were confirmed to contain the [*PSI*^+^] prion by determining that the Ade^+^ phenotype is curable by guanidine hydrochloride (data not shown), an agent known to antagonize the chaperone Hsp104 required for the propagation of the prion state of Sup35 [13]. By using semi-denaturing detergent agarose gel electrophoresis, SDD-AGE [43] we have demonstrated that overproduction of Ste18 results in the formation of detergent-resistant Ste18 polymers, as typical of yeast amyloid proteins (Figure 1A bottom). This indicates that overproduced Ste18 promotes conversion of Sup35 into the prion state, [*PSI*^+^], via producing aggregates that likely serve as heterologous nucleation centers for Sup35 aggregation. Indeed, we observed that overexpressed Ste18 fused with green fluorescent protein (GFP-Ste18) forms punctate structures that are located at the cell periphery, underlying the plasma membrane (Figure 1B). Next, we cooverexpressed GFP-Ste18 in the same cell with the Sup35 derivative, containing the Sup35NM region (composed of PrD and middle M domain) and tagged with DsRed. At high levels, the Sup35-DsRed derivative produced cytologically detectable foci, that were described previously and linked to the formation of the Sup35 prion [29,31]. Notably, a significant fraction of the Sup35-DsRed foci overlapped with the GFP-Ste18 puncta. In 20 out of 22 (91%) cells containing both fluorescently tagged proteins, colocalization of one or more of Sup35-DsRed foci with GFP-Ste18 foci was detected. Overall, 48 out of 86 (56%) Sup35-DsRed foci detected in these cells overlapped with GFP-Ste18 aggregates (Figure 1C). Partial colocalization of Sup35NM and Ste18 assemblies supports the model suggesting that Ste18 and Sup35 transiently interact during the nucleation of Sup35 prion.

### 2.2. Ste18 Aggregation and Ste18-Mediated [PSI^+^] Induction Depend on the Membrane Association, but Do not Depend on Ste18 Function in the Signaling Pathway 

Localization of the Gβγ complex to the plasma membrane after dissociation from the Gα subunit is required for signal transduction. Posttranslational modifications (PTMs), specifically Ste18 palmitoylation at amino acid (aa) residue cysteine 106 (C106) and farnesylation at aa residue cysteine 107 (C107) (Figure 1D) are essential for its association with the membrane [39,40]. Arginine residues 34 (R34) and 48 (R48) are also crucial for signal transduction and implicated in the interaction of Gβγ with their downstream intracellular effectors rather than in the anchoring of Ste18 to the membrane [44]. By using the pheromone-induced growth arrest assay, we have confirmed that each of the mutations C106S, C107S or R34K abolishes pheromone signaling, as seen by the lack of the growth arrest ring around the filter with α-factor, while mutation R48K does not affect sensitivity to pheromone (Figure 1E). C107S and especially C106S substitution although decreased the steady-state levels of the Ste18 protein, suggesting that the protein not associated with a membrane could be more proteolytically unstable. Notably, substitutions C106S and C107S also abolished aggregation of overproduced Ste18 (as detected SDD-AGE) and its ability to promote [*PSI*^+^] formation (as detected on −Ade medium), while substitutions R34K and R48K had no impact on these phenotypes (Figure 1E). As discussed below (see next section), an anti-aggregation effect of C106S or C107S substitutions can’t be explained by a decrease in protein levels, as even lower levels of Ste18 are capable of aggregating and inducing the [*PSI*^+^] prion in the other strains. Overall, these results indicate that aggregation and prion-nucleating activity of Ste18 depend on its association with a membrane, but not on its activity in the signal transduction pathway per se. 

### 2.3. Ste18 Ability to Induce Prions and Form Detergent-Resistant Aggregates Does not Depend on Association with Ste4

Peptide pheromone α-factor initiates mating-associated signaling pathway by promoting a release of the stimulatory Gβ/γ complex (Ste4-Ste18) from its inhibitory Gα subunit (Gpa1) [35]. As association of Ste18 with Ste4 is essential for its biological function, we next examined if this association is important for the Ste18 prion inducing ability. We analyzed the induction of [*PSI*^+^] prion by overexpression of Ste18 and Sup35 in a strain with *STE4* deletion (*ste4Δ*). Previously, it was demonstrated that levels of Ste18 are reduced in the strain depleted of Ste4, potentially due to decreased protein stability in the conditions when the Ste4/Ste18 complex is disassembled [45]. Indeed, overexpression of HA-Ste18 in a *ste4Δ* strain produces lower steady-state levels of Ste18 protein, compared to a wild-type strain (Figure 1F). Nevertheless, even decreased levels of wild-type Ste18, detected in the *ste4Δ* cells are sufficient for the formation of detergent-resistant polymers and for nucleation of [*PSI*^+^] (Figure 1F,G) This indicates that association with Ste4 is not essential for the aggregation and prion-inducing properties of Ste18. The fact that even a decreased amount of Ste18 protein in the *ste4Δ* strain is sufficient for the polymer formation and prion induction, and much higher levels of C106S and C107S mutant proteins expressed in a wild type (*STE4*) strain (Figure 1F left panel) are not, confirms that the lack of polymer formation and prion induction in the C106S and C107S mutants is not due to a decrease in the Ste18 protein levels but rather due to the loss of association with a membrane. Interestingly, mutant derivatives of Ste18 with substitutions C106S or C107S are not detected at all in the *ste4Δ* cells, suggesting that Ste18 is completely destabilized when it is associated with neither Ste4 nor membrane (Figure 1F).

### 2.4. The Prion-Inducing Ability of Ste18 Does not Require the N-Terminal Q-Rich Stretch and Is not Affected by Glutamine to Asparagine Substitution

**Figure 2:**
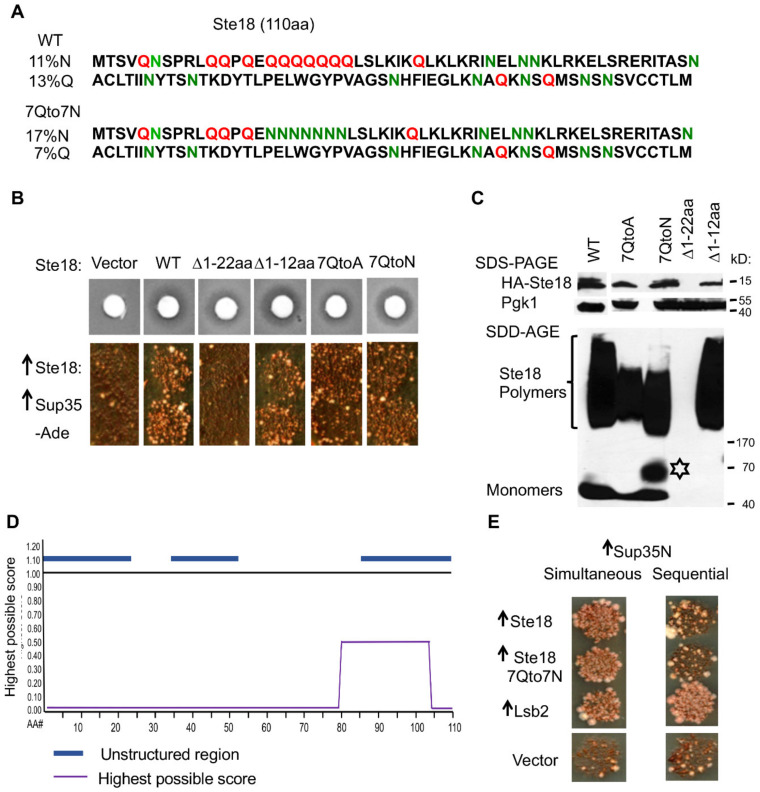
The intrinsically disordered N-proximal region of Ste18 is not essential for its prion-inducing ability. (**A**) Sequences of the wild-type Ste18 protein and its 7Qto7N variant. (**B**,**C**) Substitutions 7Qto7N or 7Qto 7A, or deletion of the first 12 aa residues of Ste18 (Δ1–12) do not abolish pheromone sensitivity (**B**), [*PSI*^+^] nucleation (**B**) and formation of detergent resistant aggregates (**C**). Deletion of the first 22 aa residues of Ste18 (Δ1–22) abolishes [*PSI*^+^] nucleation (**B**) and polymer formation (**C**), but it also greatly decreases protein levels (**C**). Phenotypes were scored and proteins were analyzed as on Figure 1A. (**D**) Among three unstructured regions (blue lines) in Ste18, only the C-terminal region shows high amyloid-forming propensity based on the highest possible score (purple line) in accordance to ArchCandy algorithm. (**E**) Comparison of [*PSI*^+^] nucleation by co-overexpression of Ste18 protein, its 7QtoN derivative, or control Lsb2 protein (expressed from *P_CUP1_* promoter) either simultaneously or sequentially with Sup35N (expressed from the *P_GAL_* promoter). For simultaneous overexpression, the *P_CUP1_* and *P_GAL_* constructs were induced simultaneously on the medium containing both 100 μM CuSO_4_ and galactose. For sequential overexpression, the *P_CUP1_* constructs were induced first on glucose medium containing 100 μM CuSO_4_, followed by replica plating onto the medium with galactose and without additional copper, where only *P_GAL_* is induced. Following induction, cultures were transferred to −Ade medium with glucose, to detect growth of [*PSI*^+^] cells. While Lsb2 promotes [*PSI*^+^] nucleation in both simultaneous and sequential induction assays, Ste18 and 7Qto7N derivatives do so only in the simultaneous (bit not in sequential) induction assay. This indicates that in contrast to Lsb2 aggregates, Ste18 aggregates are not heritable after overexpression is turned off.

PrDs and PrD-like regions of yeast proteins are frequently enriched in glutamine (Q) and/or asparagine (N) residues, as reviewed in [13]. Accordingly, aa sequence of Ste18 contains 13% of Q and 11% of N residues, with the highest concentration of Qs within the N-terminal 22 aa residues. Sequence analysis revealed that this region, predicted as unstructured, contains 11 Q and one N residue, and includes a stretch of seven Q residues (designated as 7Q stretch) at aa positions 16–22 (see Figure 2A). Deletion of the first 22 aa residues of Ste18 within the HA-Ste18 construct completely abolished [*PSI*^+^] induction (Figure 2B, column 3) and formation of detergent-resistant polymers (Figure 2C) by Ste18, however the protein containing this deletion is almost undetectable even under conditions of mRNA overexpression, suggesting either defect in translation or increased protein degradation (Figure 2C). Thus, lack of aggregation and prion induction observed with Ste18Δ1–22 protein is likely due to low protein levels in this mutant. The shorter deletion removing first 12 aa residues of Ste18 did not affect [*PSI*^+^] induction (Figure 2B, column 4), Ste18 polymerization and protein levels (Figure 2C). As this deletion still retained the 7Q stretch, we replaced respective Q residues with alanines (A) in wild-type HA-Ste18. The overproduced 7QtoA derivative of Ste18 promoted [*PSI*^+^] formation (Figure 2B, column 5) and formed detergent-resistant polymers (Figure 2C), suggesting that the N-terminal Q-rich region is dispensable for Ste18 aggregation and prion nucleation. Indeed, computational analysis of Ste18 sequence by the ArchCandy algorithm [46], capable of predicting the regions with high probability of the formation of the parallel in-register stacked intermolecular β-sheets (β-arch) that are characteristic of many amyloids, indicated that among three unstructured regions of Ste18, the C-proximal region, rather than the Q-rich N-terminus or middle region, shows high β-arch forming propensity (Figure 2A,D). Notably, none of the abovementioned alterations in Ste18 aa composition (including Δ1–22 that greatly decreases protein abundance) eliminated the sensitivity to the yeast mating pheromone (Figure 2B). Perhaps even a low level of Ste18 is sufficient for pheromone signaling, that is in an agreement with the fact that Ste18 is a low abundant protein under normal conditions (see ref. [37]). However, somewhat larger amount of protein is apparently needed for its aggregation, even though previous experiments with the *ste4Δ* strain indicate that this amount does not have to be very large.

Previously, it has been shown that in some yeast aggregating proteins containing Q-rich stretches, the replacement of Qs with Ns promotes prion propagation [47,48]. However, the replacement of 7Q stretch within N-terminal domain of Ste18 with asparagine (N) residues (7QtoN) neither affected sensitivity to pheromone nor changed the efficiency of [*PSI*^+^] induction by simultaneous overexpression of Ste18 and Sup35 (Figure 2B, column 6). While formation of detergent resistant polymers was detected in the 7QtoN variant of Ste18, the size range of polymers was shifted down, compared to wild-type Ste18 (Figure 2C). In addition, a fraction that was intermediate in size between monomers and polymers (possible dimers or other low-weight polymers) has been detected after overproduction of 7QtoN derivative of Ste18 (Figure 2C). These data indicate that QtoN substitution probably increases chaperone-dependent fragmentation of the Ste18 polymers, in analogy to some yeast prionogenic proteins observed previously [47,49] however this does not show any impact on the induction of the [*PSI*^+^] prion. Taken together, our results indicate that the QN-rich composition of the N-proximal unstructured region of Ste18 is dispensable for its aggregation and prion-inducing properties, and point to the C-proximal unstructured region as a likely determinant of these features.

### 2.5. [PSI^+^] Induction is not Associated with the Formation of Detectable Heritable Prion State by Ste18

One potential mechanism for the promotion of [*PSI*^+^] nucleation by excess Ste18 would be formation of the heritable (prion) state by the Ste18 protein itself. Indeed, other proteins promoting [*PSI*^+^] induction, such as Rnq1 and Lsb2 do so via forming a prion [17,48,50]. Even though Lsb2-based prion, [*LSB*^+^], is metastable and is lost with high frequency during cell divisions, it can be detected by using a sequential induction protocol, in which the nucleating protein is induced first, followed by the induction of Sup35 or Sup35N after the overproduction of a nucleating protein is turned off [31,48]. Thus, we tested the ability of excess Ste18 to induce formation of the Sup35 prion by using this sequential induction protocol. First, we overexpressed Ste18 from the *P_CUP1_* promoter by addition of 100 µM of Cu_2_SO_4_ to the growing yeast culture, and then moved yeast cells to the medium lacking extra Cu^2+^ but containing galactose for induction of Sup35 expression from *P_G__AL_* promoter. On such a medium, Ste18 is still produced at moderate levels due to the presence of residual 3 µM of Cu^2+^, however it is no longer overexpressed. In case if Ste18 prion inducing properties are inherited, Ste18 would be carried over to low-copper medium, leading to conversion of Sup35 into the [*PSI*^+^] prion. As shown on Figure 2E, neither wild-type Ste18 nor its 7QtoN derivative was capable of inducing conversion Sup35 into [*PSI*^+^] if Ste18 and Sup35 proteins are overexpressed sequentially (right panel). This was in contrast to Lsb2 that was able to promote nucleation of the Sup35 prion in the sequential protocol (Figure 2E) and indicates that polymers of Ste18 cannot be inherited from mother to daughter cells through several generations, at least not at the levels comparable to the Lsb2 polymers, which are themselves known to be inherited only with low frequency [48]. Therefore, Ste18 polymers do not show transmissibility in cell divisions, that is a crucial characteristic of a yeast prion. Results of this experiment show that Ste18 polymers exhibit properties similar to yeast mnemons [9]. 

### 2.6. Posttranslational Modification of Ste18 by Phosphorylation Is not Essential for its Prion Inducing Ability 

**Figure 3:**
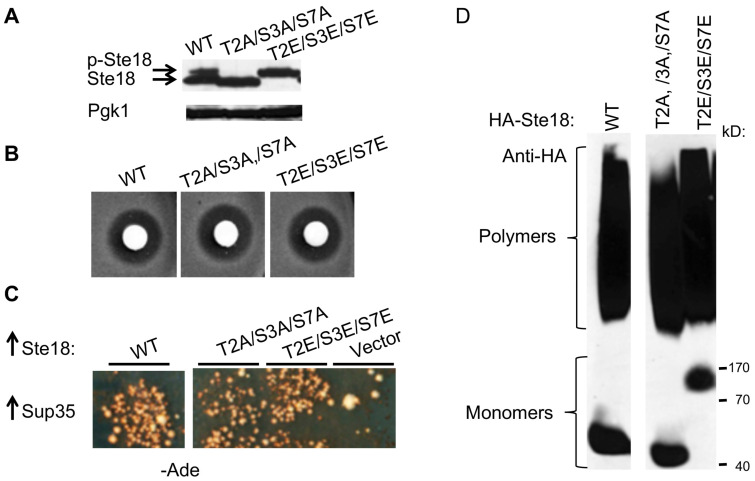
Status of Ste18 phosphorylation is not essential for prion induction. (**A**) Protein levels and mobilities in triple phospho-null mutant (T2A/S3A/S7A) or triple phosphomimetic (T2E/S3E/S7E) mutants of Ste18, in comparison to wild-type HA-Ste18 (WT) as detected by Western blot analysis. p-Ste18 is a phosphorylated (or phosphomimetic) Ste18. Pgk1 was used as a loading control. (**B**) Phospho-null and phosphomimetic mutants are responsive to pheromone based on a pheromone sensitivity agar diffusion bioassay, performed as describe in Figure 1A. (**C**,**D**) Both phospho-null and phosphomimetic mutants of Ste18 are able to nucleate [*PSI*^+^], as detected by growth on −Ade (**C**), and form detergent-resistant polymers identified by SDD-AGE gel and Western blotting with anti-HA (D).

Ste18 is phosphorylated in the N-terminal region [37]. To investigate the impact of phosphorylation on the functional and aggregation properties of Ste18, we have replaced the potentially phosphorylated residues within the N-proximal region, specifically Threonine 2 (T2), Serine 3 (S3) and Serine (S7), with either alanine (A), to abolish phosphorylation, or glutamic acid (E) to create a phosphomimetic site. As shown on Figure 3A, the triple Ste18 mutant T2A/S3A/S7A ran as a lower molecular weight band, while the triple Ste18 mutant T2E/S3E/S7E ran as a higher molecular weight band on the SDS-PAGE gel, compared to wild-type Ste18 protein. This suggests that wild-type Ste18 is partially phosphorylated, while mutations are either abolishing phosphorylation (in case of a substitution to A) or mimic complete phosphorylation (in case of a substitution to E). Neither substitution affected sensitivity to pheromone (Figure 3B), or the ability to nucleate the [*PSI*^+^] prion (Figure 3C) and form detergent resistant polymers (Figure 3D), showing that the phosphorylation of Ste18 influences neither its functionality nor aggregation and prion-inducing properties.

### 2.7. Ste18 is Ubiquitinated and Degraded in a Proteasome-Dependent Fashion

**Figure 4:**
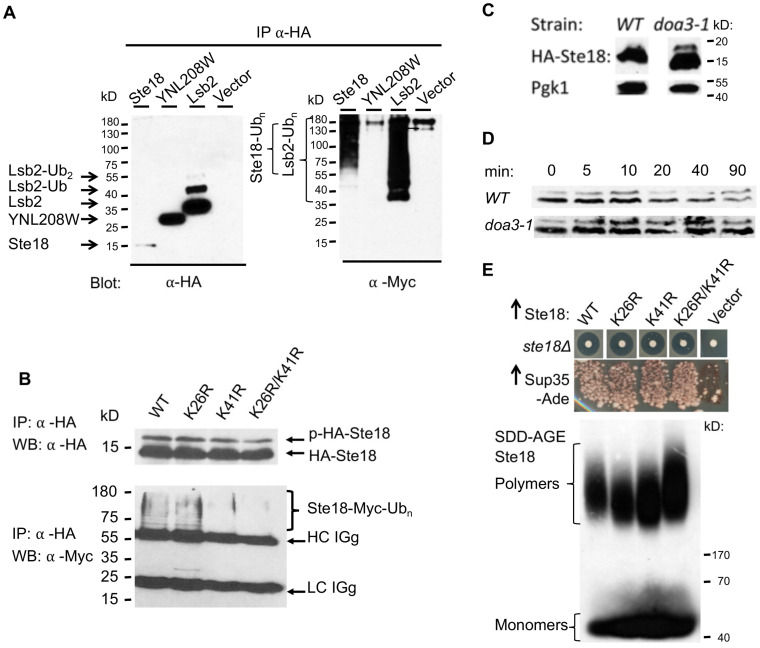
Ste18 is ubiquitinated and degraded in a proteasome dependent fashion. (**A**) Detection of Ste18 ubiquitination. Either HA-Ste18 or a protein used as a positive (HA-Lsb2) or negative (HA-YNL208W) control for ubiquitination were co-expressed with Myc-Ub. HA-tagged proteins were coimmunoprecipitated from cell extracts using anti-HA antibody (Ab) conjugated beads. The purified samples were analyzed by immunoblotting with anti-HA Ab (left panel). A band of 12.6 kD was detected in cell extract expressing Ste18, and 20.2 kD band was detected in cell extract expressing HA-YNL208W. HA-Lsb2 was presented by the major band of 23.5 kD, and two bands above it, representing mono- and di-ubiquitinated derivatives. High molecular weight conjugates representing ubiquitinated form (right panel) were detected with anti-Myc antibody in strains expressing Ste18 and Lsb2, but not in the strain expressing YNL208W. (**B**) Identification of Ste18 ubiquitination sites. The *ste18Δ* strain was co-transformed with the plasmid expressing either wild-type HA-Ste18 (WT), or K-to-R mutants as indicated, and with the plasmid expressing Myc-Ub. Wild-type or mutant HA-Ste18 protein was co-IPed from cell extracts using anti-HA Ab conjugated beads, and resulting samples were analyzed by immunoblotting with anti-HA Ab (top panel) and anti-Myc Ab (bottom panel). Ste18 proteins are represented by non-phosphorylated (bottom) and phosphorylated (top) HA-reactive bands (see Figure 3A), while high molecular weight conjugates representing ubiquitinated Ste18 are detected by anti-Myc antibody. Ubiquitination is seen for the wild-type type and K26R Ste18, but not for K41R and double K26R/K41R Ste18 derivatives. (**C**,**D**) Ste18 degradation is affected by the *doa3-1* mutation. HA-Ste18 was expressed at low levels from *P_CUP1_* in the wild-type and proteasome deficient *doa3-1* strains. The steady state levels (**C**) and half-life (**D**) of Ste18 were measured. For half-life measurements by cycloheximide chase, cells were grown to log phase, and total protein lysates were prepared immediately or at the indicated time point after the addition of cycloheximide to a final concentration of 250 µg/mL to arrests protein synthesis. Lysates were fractionated by SDS-PAGE, followed by Western blotting and reaction to anti-HA Ab. (**E**) Lack of Ste18 ubiquitination affects neither response to pheromone nor [*PSI*^+^] nucleation and formation of detergent-resistant polymers. The *ste18Δ* strain was transformed with plasmids expressing either wild-type HA-Ste18 (WT), or K-to-R mutants as indicated, from *P_CUP1_* promoter. Pheromone sensitivity of *ste18Δ* derivatives expressing wild type or mutant Ste18 was assessed by the agar diffusion (halo) bioassay, [*PSI*^+^] induction was detected by growth on −Ade medium, and formation of detergent-resistant polymers was detected by SDD-AGE as described in Figure 1A.

Ste18 was detected as ubiquitinated protein in a large-scale MS analysis [41], but to our knowledge, this was never followed up in detailed biochemical studies. Given that ubiquitination and protein degradation are also known to modulate prion-like aggregation [13,51,52], we next investigated ubiquitination and proteasome-dependent degradation of Ste18. To detect ubiquitination of Ste18, we co-expressed HA-Ste18 and Myc-tagged Ub (Myc-Ub) in *ste18Δ* cells. HA-Lsb2 and HA-YNL208W proteins respectively served as a positive and negative controls for ubiquitination. Proteins were immunoprecipitated (IPed) using anti-HA antibody and analyzed by western blotting. Immunoblotting with anti-HA antibodies revealed the 12.6 kD HA-Ste18, 23.5 kD HA-Lsb2 and 20.2 kD HA-YNL208W bands (Figure 4A, left panel). In case of Lsb2, additional HA-positive bands that migrated above Lsb2 protein were detected; based on our previous studies, these bands represent mono and di-ubiquitinated HA-Lsb2 [31]. Such mono- and di-ubiquitinated bands were not observed for Ste18. To visualize ubiquitinated forms at higher sensitivity level, we probed IPed proteins with anti-Myc antibodies that recognize Myc-fused Ub (Figure 4A right panel). High molecular weight (MW) smear was detected by anti-Myc antibodies in samples derived from cells expressing HA-Ste18 and HA-Lsb2, but not in cells expressing HA-YNL208W. These data confirm that similar to Lsb2, Ste18 is ubiquitinated, and high molecular weight of the ubiquitinated bands co-IPed with Ste18 points to its polyubiquitination. 

In the previous large-scale analysis of ubiquitinated proteins, the potential Ste18 ubiquitination sites were identified as lysine residues 26 (K26) and 41 (K41) [41]. To check these data, we generated the plasmid-borne *HA-STE18* constructs with K26R, K41R and double K26R/K41R substitutions under control of the *P_CUP1_* promoter, and co-expressed each of them with the plasmid producing Myc-tagged Ub (Myc-Ub) in *ste18Δ* cells. The wild-type *HA-STE18* construct was used as a control. Immunoblotting with anti-HA antibodies detected the 12.6 kD HA-Ste18 bands in all cultures after induction of Ste18 expression by addition of CuSO_4_ (Figure 4B, top panel). Additional HA-positive bands that migrated above HA-Ste18 band and likely represented phosphorylated forms of Ste18 were also evident in all samples (Figure 4B, top panel). To visualize ubiquitinated forms of Ste18 and its K-to-R mutants, we probed immunoprecipitated proteins with anti-Myc antibodies recognizing Myc-Ub. Higher molecular weight (MW) corresponding to polyubiquitinated Ste18 species smear was detected by anti-Myc antibodies in samples derived from cells expressing wild-type and K26R mutant of Ste18, but not in K41R and K26R/K41R mutants (Figure 4B, bottom panel). These data indicate that K41 rather than K26 residue is a primary ubiquitination site of Ste18. In addition, we demonstrated that the steady state levels of Ste18 are increased in the *doa3-1* strain, where the proteasomal activity is impaired [53], compared to proteasome-active wild-type cells (Figure 4C). To corroborate these data further, we performed the cycloheximide chase experiment [54] as shown on Figure 4D, and detected the decrease in the levels of both unmodified and phosphorylated forms of Ste18 over time in wild-type cells, while they remained stable after 90 min in the proteasome-deficient *doa3-1* strain (Figure 4D). To check if ubiquitination influences aggregation and prion-inducing properties of Ste18 and its functioning in the signaling pathway, we examined the effects of K26R, K41R and K26R/K41R substitutions on the formation of detergent resistant aggregates, nucleation of [*PSI*^+^] and sensitivity to pheromone. None of these characteristics was altered by respective mutations (Figure 4E), implying that while lack of ubiquitination antagonizes Ste18 degradation, it does not directly affect its aggregation or biological properties per se.

## 3. Discussion

### 3.1. Prion Inducing Properties of Ste18

Ste18 was identified as one of several Q/N rich proteins that can trigger conversion of Sup35 into prion [PSI^+^] when both proteins are co-overproduced in yeast [17]. We confirmed this observation and demonstrate that Ste18 forms SDS-resistant polymers (a hallmark of amyloids) when present at high levels (Figure 1A). This suggests that the potential mechanism behind the Ste18 ability to induce [PSI^+^] formation is providing the initial nucleus, or “seed” for Sup35 aggregation. Our data demonstrating that some Ste18 and Sup35 aggregates are colocalized in the cells undergoing [PSI^+^] induction (Figure 1C and Supplementary Movie S1) support this notion. Possibly (like in case of Rnq1), colocalization with Ste18 is no longer maintained after Sup35 itself is converted into an amyloid state [19]. Dynamics of Ste18-Sup35 interaction could be of interest for future investigations.

Many yeast prion-forming proteins contain intrinsically disordered regions (IDRs), also termed “Prion Domain Like Domains” (PrDL) domains [13,55], that are similar to the Sup35 prion domain (PrD) in aa composition (for example, high N and/or Q contents) and are thought to be responsible for both prion formation by these proteins and their interactions with Sup35 PrD during heterologous cross-seeding. N-termini of all known Gγ subunits including Ste18 exhibit a high degree of intrinsic disorder [37]. Surprisingly, we found that Ste18 prion inducing ability does not depend on its N-terminal Q-rich IDR, since neither deletion of the first 12 aa residues nor replacement of 7Q stretch (residues 16–22) by alanines abolished the ability of Ste18 to aggregate and nucleate the [PSI^+^] prion (Figure 2B,C). Notably, Ste18 was not identified as a protein with PrDL by a hidden Markov model (HMM)-based approach used to predict potential yeast prions [55]. However, computational analysis using the ArchCandy algorithm, developed later and capable of predicting the regions with high probability of formation of the parallel in-register stacked intermolecular β-sheets (β-archs), characteristic of many amyloids [46,56,57], uncovers that the C-proximal region of Ste18, rather than the Q-rich N-terminus, constitutes a region with high β-arch forming propensity (Figure 2D). This C-terminal region also overlaps with one of three IDRs found in Ste18, however it is not QN-rich. Examples of non-QN rich domains with prion or prion-like properties are known from both mammalian (e.g., PrP) and fungal (Het-s of *Podospora anserina* and Mod5 of *Saccharomyces cerevisiae*) systems [8,58,59]. 

Some other proteins shown to promote initial nucleation of the Sup35 prion, e.g. Rnq1, Mod5 and Lsb2 are also capable of forming a heritable (prion) state on their own [17,48,60,61]. In contrast to these proteins, [*PSI*^+^]-inducing aggregates of Ste18 are not heritable (Figure 2E), at least not at the level comparable to aggregated Lsb2, which is itself a metastable prion, transmissible only at low frequency [48]. It is not clear now whether non-heritability of Ste18 could be related to its high proteolytic turnover and calls for further investigations. Lsb2 is also efficiently degraded via UPS when it is present in a non-prion form, however aggregates would likely need to be disassembled prior to proteolysis by a proteasome. In any case, Ste18 behavior makes it different from bona fide prions. Rather, Ste18 aggregates resemble recently described protein assemblies termed mnemons [9], which are based on the same molecular mechanisms as prions, however tend to stay in the mother cell and are not inherited by daughters. Notably, the first mnemon found in yeast cells is formed by Whi3 protein, that is also involved in the mating signaling pathway, however at later stages than Ste18 [9,62]. 

### 3.2. Role of the Association of Ste18 with a Membrane in its Aggregation and Prion-Inducing Properties

It has been reported that interactions with membranous compartments and/or membrane-associated cytoskeletal structures modulate formation of prions or prion-like aggregates by some proteins. Conflicting data point to either stimulatory or inhibitory effects of the lipid and protein environment in the plasma membrane in the conversion of membrane-associated protein PrP (linked to transmissible spongiform encephalopathies) into a prion [63]. Alteration of the integrity of the detergent-resistant membrane components, so-called lipid rafts in neuronal cells also promoted misfolding of the PrP-related protein Shadoo [64]. At the initial stages of de novo formation of the yeast prion [*PSI*^+^] at high levels of Sup35 or Sup35N in the [*PIN*^+^] strain, fluorophore-tagged Sup35 aggregates can be visualized as a mesh of rings along the inner cell membrane [29,65,66]. It was suggested that the first step in the de novo prion induction is the formation of a prion “seed” at the cell periphery, followed by a stage in which this seed grows bidirectionally into a peripheral filament or “line” by the addition of nonprion conformers to both ends of the seed. The role of pre-existing aggregates of heterologous PrDL-containing proteins in this process could be to aid in the formation of the initial prion seed, while the localization of initial seeds and seed-derived filaments near the membrane could be due to the fact that the heterologous cross-seeding proteins are present in the assemblies associated with the membrane. Such assemblies may immobilize overexpressed Sup35, thereby promoting initial prion nucleation. Previous data from us and others indicate that proteins associated with cortical actin patches modulate nucleation of the Sup35 prion and formation of filamentous aggregates in the [*PIN*^+^] background [29,65,66], while Lsb2 protein is required to be associated with the cortical actin cytoskeleton via Las17 for the promotion of [*PSI*^+^] formation in the absence of Rnq1 prion [31]. Therefore, the sub-membrane actin cytoskeletal structures generate sites for the initial formation of the Sup35 prion (and possibly other prions). 

While there are no data showing association of Ste18 with the actin cytoskeleton, Ste18 is associated with cell membrane via a farnesyl-directing CaaX box motif, encompassing the C-terminal residues from 107 to 110 [67]. The farnesyl group is transferred to the cysteine residue in the CaaX box motif of the Ste18 precursor, followed by proteolytic removal of the last three residues (108–110) and methylation. The C106 residue located nearby is a site for palmitoylation, that is also involved in the association of Ste18 with a membrane [39,40]. Mutation of either C106 or C107 eliminates association with a membrane. We have shown that the replacement of either C106 or C107 with a serine (S) residue, knocking out the palmitoylation or farnesylation, respectively, and therefore abolishing the Ste18 association with a membrane, also entirely abolishes the ability of Ste18 to form detergent resistant aggregates and promote nucleation of the Sup35 prion (Figure 1E). As both C106 and C107 residues are located outside of the β-arcade region identified by Arch Candy algorithm (Figure 2D), and neither substitution influences the predicted propensity to form β-archs, this result indicates that the association with a membrane is required for the Ste18 aggregation and prion-inducing properties. 

In all Gβγ complexes the Gγ subunit serves as a membrane anchor while Gβ actually binds to each of the effectors, using interaction surfaces that were buried when it was associated with Gα. When released from Gα, the Gβγ complex Ste4/Ste18 helps to recruit scaffolding protein Ste5 and its associated MAP kinase cascade components to the membrane, thus promoting transduction of the pheromone signal. It is known that the Ste18 protein is barely detectable in the *ste4Δ* mutant due to an increased rate of protein turnover [45]. Indeed, we observed that even though Ste18 was overexpressed in the *ste4Δ* strain, its levels were considerably lower when compared to wild-type strain (Figure 1F). However, even at these lower levels, Ste18 was able to polymerize and induce formation of the Sup35 prion (Figure 1F,G). Mutations at the N-proximal region of Ste18 that are known to disrupt the pheromone signaling pathway without affecting the membrane association of Ste18 [44] affected neither Ste18 protein levels nor its ability to aggregate and induce Sup35 prion (Figure 1E). These data imply that neither functionality of Ste18 in the signaling pathway nor its interactions with Ste4 or downstream components of the pathway play role in the aggregation and prion-inducing properties of Ste18. Rather, association of Ste18 with a membrane determines both its functional and prion-inducing properties. 

### 3.3. Post-Translational Modifications Modulate Levels of Ste18 but not its Aggregation and Prion-Inducing Abilities

Protein phosphorylation is one of the major classes of reversible PTMs [68], and it has been suggested to play a role in the regulation of self-association and pathogenic aggregation of some proteins such as tau [69] and Aβ [70], associated with Alzheimer’s disease, and TDP-43 [71,72] and FUS [73], associated with some forms of Amyotrophic lateral sclerosis. Eukaryotic Gγ subunits, including yeast Gγ protein Ste18 harbor at least two (in case of Ste18, three) phosphorylation sites located within their N-terminal IDRs [37]. It is shown that Ste18 phosphorylation is rapidly activated and then further increased during exposure to the mating pheromone [37]. Phosphorylation is essential for the maintenance of steady state protein levels of Ste18, and is specifically linked to about threefold increase in the abundance of Ste18 protein in the presence of pheromone [37]. However, mutations either destroying or mimicking phosphorylation at three potential sites, previously identified within the N-terminal region of Ste18 [37,74] did not show any effect on aggregation and prion inducing abilities of overexpressed Ste18 (Figure 3C,D). This indicates that phosphorylation per se is not directly involved in the regulation of the aggregation and prion-inducing properties of Ste18, although it is possible that phosphorylation may influence these processes indirectly by modulating Ste18 levels under physiological conditions. Further studies might clarify this question.

Another PTM that may be involved in the regulation of the pheromone signaling pathway is ubiquitination [75]. Ste18 was detected as a ubiquitinated protein in a large-scale MS analysis [41], with positions K26 and K41 identified as potential ubiquitination sites. Here we demonstrated that under normal growth conditions, K41 is the major Ste18 ubiquitination site, and that Ste18 is a short-lived protein with a half-life of about 20 min, whose levels are controlled by UPS. Like phosphorylation, Ste18 ubiquitination does not have any direct effect on the aggregation and prion-inducing properties of overproduced Ste18 (Figure 4E), although it is possible that at normal physiological conditions, ubiquitination may influence these properties via modulating Ste18 abundance. 

### 3.4. Protein Aggregation Based Memory of Environmental and Physiological Stimuli

As our experiments have demonstrated that Ste18 protein produces detergent-resistant aggregates at high levels, one could argue that such a property might have certain biological implications. Indeed, signal transduction pathways are ubiquitous mechanisms that allow cells to respond appropriately to intracellular and environmental cues. This process requires tuned regulation which is achieved by formation of different protein assemblies at specific cellular compartments. PrDL-mediated protein assemblies can act as mnemons [76], holding cellular memory about certain physiological events, and/or trigger formation of self-perpetuating heritable aggregates (prions) by the same or other proteins, which may “replicate” such memory in cell generations. Examples of aggregate-based cellular memory have been described in various organisms [9], including the role of self-perpetuating protein polymers in the long term memory of behavioral patterns in higher eukaryotes [77,78].

Several proteins involved in the yeast pheromone signaling pathway contain PrDL regions. As mentioned above, the PrDL-dependent assemblies that control cellular memory have been described for one of these proteins, namely Whi3 [9]. During exposure to pheromone, Whi3 captures and represses translation of one of the cycline (specifically, Cln3) mRNA, thus inhibiting G1/S phase transition and leading to G1 arrest, a pre-requisite for mating. However, if mating does not occur, yeast cells eventually escape the G1 arrest and resume mitotic divisions (budding); moreover, these cells are no longer arrested after the exposure to pheromone in the future, although this insensitivity to pheromone arrest is not transmitted to their daughters. The mechanism behind pheromone insensitivity is the formation of a super-assembly (aggregate) of Whi3, which is accumulated and retained in the mother cells for a long period of time. It is also shown that Whi3 assemblies are formed in aged yeast cells, making them incapable of mating [62]. At the molecular level, Whi3 super-assemblies are similar to yeast prions as they are driven by QN-rich PrDL domain. Whi3 assembly represents an example of mnemon, acting as a device for maintaining the cellular memory of the exposure to pheromone.

As Ste18 acts at an earlier stage of the same pheromone signaling pathway as does Whi3, we suggest that Ste18 also might potentially either be involved in cellular memory itself, or promote mnemon formation by other protein(s). In response to pheromone, the Ste4-Ste18 dimer nucleates the formation of a multiprotein complex [34,35,36], that triggers mitogen-activated protein kinase cascade [79], resulting in G1 cell cycle arrest [80] via Inhibition of Cln-Cdc28 activity, repression of transcription and promotion of turnover of cyclins Cln1 and Cln2 [80]. As levels of Ste18 are regulated by PTMs (see above) and are increased in response to pheromone [37], it is possible that such an increase may trigger formation of Ste18 aggregates in a fraction of the cells. At this point, it is hard to say what impact (if any) would such an aggregation have on the mating process. As Ste18 polymerization is likely to be much less frequent in natural conditions than in case of artificial Ste18 overproduction, and its phenotypic consequences are not known, it is difficult to detect the naturally formed Ste18 aggregates directly at the moment, and further studies are needed to test this hypothesis and decipher the biological consequences of Ste18 aggregation. Based on our results we envision a strong possibility that, similar to Whi3, the Ste18 aggregated assemblies would not be inherited by daughter cells and may therefore represent another mother-specific mnemon involved in mating-related signaling. However, based on the ability of Ste18 assemblies to nucleate the Sup35 prion, it is possible these assemblies are capable of cross-seeding formation of heritable aggregates by other proteins too.

Notably, the G protein signaling pathway also involves another branch that leads to repolarization of growth in the direction of the mating partner in response to pheromone, leading to the formation of projectiles (“shmoo”) and ultimately to cell fusion [81]. In this branch, Gβγ recruits the polarity proteins Far1 and Cdc24 to the regions of plasma membrane receiving the highest pheromone dose [82,83]. This leads to redistribution of cortical actin patches resulting in polarized growth along the gradient of pheromone [84,85]. Major actin nucleation regulators are Las17 protein (WASP) [86] and Las17 binding proteins, including the stress-inducible protein Lsb2 [31]. We have shown previously that like Ste18, overproduced Lsb2 forms detergent resistant aggregates, capable of inducing conversion of Sup35 protein into a [*PSI^+^*] prion [31]. As in the case of Ste18, aggregation and prion-inducing patterns of Lsb2 are strictly dependent on its intracellular localization (in particular, on its association with the cortical actin patches via Las17). Moreover, we have shown that Lsb2 can acquire a [*PSI^+^*]-inducing state in response to thermal stress, thus generating a mechanism for maintaining a memory of stress [48,87]. In contrast to Ste18, this state of Lsb2 can be considered as a metastable prion, as it can be transmitted to daughter cells, albeit with low frequency. Our data show that fluorophore-tagged Lsb2 exhibits polarized distribution during mitosis [31] and is observed in the tip of mating protrusion when cells were treated with pheromone (Supplementary Figure S1). Considering the significance of actin polarization during mating and the understanding that signaling pathways regulating cell fate decisions should be interconnected to varying degrees [88], it would be interesting to check if “pheromone memory” hypothetically mediated by aggregation of Ste18, and “stress memory”, mediated by aggregation of Lsb2, could be interconnected and/or involved in Ste18′s cross-talk, when yeast cells are undergoing the mating process during or after stress.

Importantly, the Gβγ complex represents a conserved signaling pathway, heavily involved in various signaling pathways in higher eukaryotes [89]. Moreover, the development of cell polarity in response to external stimuli is a feature of most eukaryotic cells [90]. This indicates that potential “memory” capabilities of some proteins involved in these processes may apply to organisms other than yeast (including humans).

## 4. Materials and Methods

### 4.1. Strains and Plasmids

Saccharomyces cerevisiae strains used in this study are listed in Table 1.

Disruptions of chromosomal genes were generated by PCR-mediated gene replacement [92] and verified by sequencing. The *STE18* coding sequence under the copper inducible promoter *P_CUP1_* was placed into the centromeric *URA3* plasmid pRS316 [93]. Mutations at *STE18* and a fusion with GFP were generated by the overlapping PCR cloning approach. Other plasmids used in this study included: the centromeric *TRP1* plasmid pFL39-CEN-GAL-Sup35N [52] employed for [*PSI*^+^] induction assay; 2µ DNA based *TRP1* plasmid pYEp105-Myc-Ub, employed for the analysis of Ste18 ubiquitination [91]; and the centromeric *LEU2* plasmid pRS315-Sup35-dsRed [94], used for the detection of Sup35 aggregation.

### 4.2. Growth Conditions and Phenotype Detection

Standard yeast media, cultivation conditions, procedures for transformation and phenotype scoring were used [95]. The presence of [*PSI*^+^] was monitored by its ability to suppress the reporter *ade1-14* (containing the premature stop codon, *UGA*), resulting in the growth on the medium lacking adenine (−Ade) [42]. Plates are usually scanned after 10 days of incubation. An agar diffusion bioassay (halo assay) for the detection of the response to α-factor-induced cell cycle arrest was performed as described [96]. Specifically, cells were embedded in SD-Ura top agar, and a filter disk with 15 μg of α-factor (Sigma-Aldrich, St. Louis, MO, USA) were placed on the surface. Response to pheromone was visualized as a halo of growth arrest after 24 h of incubation. In all growth experiments, typically 12 or more transformants were analyzed per each strain/plasmid combination; usually the vast majority of them did show the same result.

### 4.3. Protein Isolation and Analysis

Cells were lysed either by boiling in the SDS-containing loading buffer, or by vortexing with glass beads in case of immunoprecipitation (IP) experiments [31]. Protein were run on SDS-PAGE gel, or (in case of analysis of protein polymer), on semi-denaturing detergent agarose gel electrophoresis (SDD-AGE) gel [97,98,99]. Positions of molecular weight markers for SDS-PAGE or SDD-AGE gels are shown on figures; it should be noted that the correspondence of the monomer positions to markers is grossly imprecise on SDD-AGE gels due to high diffusion. Anti-HA-agarose (Thermo Fisher Scientific, Waltman, MA, USA) was used for IP. The cycloheximide chase experiment was performed as described [31]. An equal number of cells was collected at the specific time point and lysed by boiling to isolate protein. Proteins in extracts or immunoprecipitates were detected by Western analysis followed by reaction to specific antibodies, such as: anti-HA HA.11 (Covance, Inc., Emerville, CA, USA); anti-Myc 9B11 (Cell Signaling Technology, Danvers, MA, USA); anti-Pgk (Molecular Probes, Inc., Eugene, OR, USA). In all experiments, we used appropriate secondary antibodies from GE Healthcare Chicago, IL, USA. 

### 4.4. Fluorescence Microscopy 

Live cells with GFP-Ste18 were imaged with a 100× oil immersion objective on the Olympus IX81 microscope (Olympus America, Inc., Melville, NY), equipped with a Hamamatsu digital camera (Hamamatsu Photomics, Japan). Live cells with GFP-Ste18 and Sup35NM-dsRED were imaged on Delta Vision Core system (Applied Precision / GE Healthcare, Issaquah, WA, USA) consisting of Olympus IX70 inverted microscope (Olympus America, Inc. Melville, NY, USA) with 100× NA 1.4 oil immersion objective and a CoolSnap HQ 12-bit camera (Photometrics, Tucson, AZ, USA) controlled by SoftWoRX software (GE Healthcare, Chicago, IL, USA). Filters used for imaging were FITC (Ex 490/20; Em 528/38), RD-TR (Ex 555/28; Em 617/73) of the 86000 Sedat Quadruple Filter Set (Chroma Technology Corp, Bellows Falls, VT). Z-stacks of 21 focal planes were acquired with a step size of 350 nm and XY pixel size of 65 nm. Exposure was set to 25 ms for RD-TR channel, and for 80 ms for FITC channel. Movie sequences were deconvolved with SoftWoRx, scaled manually to 8 bit using linear LUT and the same range of scaling for all the images. Scaled images were overlaid. Overlay images were converted into avi movies in Imaris (Bitplane/Oxford Instruments, South Windsor, CT, USA).

## Figures and Tables

**Table 1:** Yeast strains.

Strain	Genotype	Source
GT409 (WTY222)	*MAT*a *ade1–14 (UGA) his3-∆200 leu2–3,112 lys2–801 trp1 ura3–52* [*psi^−^ pin^−^*]	[51]
WTY775	*ste18Δ::kanMX6* disruption in GT409	This study
WTY770	*ste4Δ::kanMX6* disruption in GT409	This study
MHY501	*MAT*α *his3-Δ200 leu2-3,112 lys2-801 trp1 ura3-52*	[91]
MHY3646	*doa3-1* derivative of MHY501	[91]

## Supplementary Materials

Supplemental Information includes one figure and one movie, and can be found at https://www.mdpi.com/1422-0067/21/14/5038/s1.

## References

[1] Prusiner S.B. (1998). Prions. Proc. Natl. Acad. Sci. USA.

[2] Wickner R.B. (2016). Yeast and Fungal Prions. Cold Spring Harb. Perspect. Biol..

[3] McGlinchey R.P., Kryndushkin D., Wickner R.B. (2011). Suicidal [PSI+] is a lethal yeast prion. Proc. Natl. Acad. Sci. USA.

[4] Halfmann R., Jarosz D.F., Jones S.K., Chang A., Lancaster A.K., Lindquist S. (2012). Prions are a common mechanism for phenotypic inheritance in wild yeasts. Nature.

[5] Jarosz D.F., Brown J.C.S., Walker G.A., Datta M.S., Ung W.L., Lancaster A.K., Rotem A., Chang A., Newby G.A., Weitz D.A. (2014). Cross-kingdom chemical communication drives a heritable, mutually beneficial prion-based transformation of metabolism. Cell.

[6] Jarosz D.F., Lancaster A.K., Brown J.C., Lindquist S. (2014). An evolutionarily conserved prion-like element converts wild fungi from metabolic specialists to generalists. Cell.

[7] Newby G.A., Kayatekin C. (2018). Microbial specialization by prions. Prion.

[8] Saupe S.J. (2007). A short history of small s: A prion of the fungus *Podospora anserina*. Prion.

[9] Caudron F., Barral Y. (2013). A super-assembly of Whi3 encodes memory of deceptive encounters by single cells during yeast courtship. Cell.

[10] Liebman S.W., Chernoff Y.O. (2012). Prions in yeast. Genetics.

[11] Tuite M.F., Serio T.R. (2010). The prion hypothesis: From biological anomaly to basic regulatory mechanism. Nat. Rev. Mol. Cell Biol..

[12] Killian A.N., Miller S.C., Hines J.K. (2019). Impact of Amyloid Polymorphism on Prion-Chaperone Interactions in Yeast. Viruses.

[13] Chernova T.A., Wilkinson K.D., Chernoff Y.O. (2017). Prions, Chaperones, and Proteostasis in Yeast. Cold Spring Harb. Perspect. Biol..

[14] Chernova T.A., Wilkinson K.D., Chernoff Y.O. (2014). Physiological and environmental control of yeast prions. FEMS Microbiol. Rev..

[15] Chernova T.A., Chernoff Y.O., Wilkinson K.D. (2019). Yeast Models for Amyloids and Prions: Environmental. Modul. Drug Discov. Mol..

[16] Tuite M.F. (2013). The natural history of yeast prions. Adv. Appl. Microbiol..

[17] Derkatch I.L., Bradley M.E., Hong J.Y., Liebman S.W. (2001). Prions affect the appearance of other prions: The story of [PIN(+)]. Cell.

[18] Osherovich L.Z., Weissman J.S. (2001). Multiple Gln/Asn-rich prion domains confer susceptibility to induction of the yeast [PSI(+)] prion. Cell.

[19] Derkatch I.L., Uptain S.M., Outeiro T.F., Krishnan R., Lindquist S.L., Liebman S.W. (2004). Effects of Q/N-rich, polyQ, and non-polyQ amyloids on the de novo formation of the [PSI+] prion in yeast and aggregation of Sup35 in vitro. Proc. Natl. Acad. Sci. USA.

[20] Yang Z., Hong J.Y., Derkatch I.L., Liebman S.W. (2013). Heterologous gln/asn-rich proteins impede the propagation of yeast prions by altering chaperone availability. PLoS Genet..

[21] Li X., Rayman J.B., Kandel E.R., Derkatch I.L. (2014). Functional role of Tia1/Pub1 and Sup35 prion domains: Directing protein synthesis machinery to the tubulin cytoskeleton. Mol. Cell.

[22] Gokhale K.C., Newnam G.P., Sherman M.Y., Chernoff Y.O. (2005). Modulation of prion-dependent polyglutamine aggregation and toxicity by chaperone proteins in the yeast model. J. Biol. Chem..

[23] Gong H., Romanova N.V., Allen K.D., Chandramowlishwaran P., Gokhale K., Newnam G.P., Mieczkowski P., Sherman M.Y., Chernoff Y.O. (2012). Polyglutamine toxicity is controlled by prion composition and gene dosage in yeast. PLoS Genet..

[24] Meriin A.B., Zhang X., He X., Newnam G.P., Chernoff Y.O., Sherman M.Y. (2002). Huntington toxicity in yeast model depends on polyglutamine aggregation mediated by a prion-like protein Rnq1. J. Cell Biol..

[25] Jucker M., Walker L.C. (2011). Pathogenic protein seeding in Alzheimer disease and other neurodegenerative disorders. Ann. Neurol..

[26] Walker L.C., LeVine H. (2012). Corruption and spread of pathogenic proteins in neurodegenerative diseases. J. Biol. Chem..

[27] Liu B., Larsson L., Caballero A., Hao X., Oling D., Grantham J., Nystrom T. (2010). The polarisome is required for segregation and retrograde transport of protein aggregates. Cell.

[28] Toret C.P., Drubin D.G. (2006). The budding yeast endocytic pathway. J. Cell Sci..

[29] Ganusova E.E., Ozolins L.N., Bhagat S., Newnam G.P., Wegrzyn R.D., Sherman M.Y., Chernoff Y.O. (2006). Modulation of prion formation, aggregation, and toxicity by the actin cytoskeleton in yeast. Mol. Cell. Biol..

[30] Bailleul P.A., Newnam G.P., Steenbergen J.N., Chernoff Y.O. (1999). Genetic study of interactions between the cytoskeletal assembly protein sla1 and prion-forming domain of the release factor Sup35 (eRF3) in *Saccharomyces cerevisiae*. Genetics.

[31] Chernova T.A., Romanyuk A.V., Karpova T.S., Shanks J.R., Ali M., Moffatt N., Howie R.L., O’Dell A., McNally J.G., Liebman S.W. (2011). Prion induction by the short-lived, stress-induced protein Lsb2 is regulated by ubiquitination and association with the actin cytoskeleton. Mol. Cell.

[32] Dowell S.J., Brown A.J. (2009). Yeast assays for G protein-coupled receptors. Methods Mol. Biol..

[33] Cattaneo F., Guerra G., Parisi M., De Marinis M., Tafuri D., Cinelli M., Ammendola R. (2014). Cell-Surface Receptors Transactivation Mediated by G Protein-Coupled Receptors. Int. J. Mol. Sci..

[34] Dohlman H.G., Thorner J.W. (2001). Regulation of G protein-initiated signal transduction in yeast: Paradigms and principles. Annu. Rev. Biochem..

[35] Alvaro C.G., O’Donnell A.F., Prosser D.C., Augustine A.A., Goldman A., Brodsky J.L., Cyert M.S., Wendland B., Thorner J. (2014). Specific alpha-arrestins negatively regulate Saccharomyces cerevisiae pheromone response by down-modulating the G-protein-coupled receptor Ste2. Mol. Cell. Biol..

[36] Arkowitz R.A. (2009). Chemical gradients and chemotropism in yeast. Cold Spring Harb. Perspect. Biol..

[37] Dewhurst H.M., Choudhury S., Torres M.P. (2015). Structural Analysis of PTM Hotspots (SAPH-ire)--A Quantitative Informatics Method Enabling the Discovery of Novel Regulatory Elements in Protein Families. Mol. Cell Proteomics.

[38] Choudhury S., Baradaran-Mashinchi P., Torres M.P. (2018). Negative Feedback Phosphorylation of Ggamma Subunit Ste18 and the Ste5 Scaffold Synergistically Regulates MAPK Activation in Yeast. Cell Rep..

[39] Hirschman J.E., Jenness D.D. (1999). Dual lipid modification of the yeast ggamma subunit Ste18p determines membrane localization of Gbetagamma. Mol. Cell. Biol..

[40] Manahan C.L., Patnana M., Blumer K.J., Linder M.E. (2000). Dual lipid modification motifs in G(alpha) and G(gamma) subunits are required for full activity of the pheromone response pathway in Saccharomyces cerevisiae. Mol. Biol. Cell.

[41] Swaney D.L., Beltrao P., Starita L., Guo A., Rush J., Fields S., Krogan N.J., Villen J. (2013). Global analysis of phosphorylation and ubiquitylation cross-talk in protein degradation. Nat. Methods.

[42] Chernoff Y.O., Uptain S.M., Lindquist S.L. (2002). Analysis of prion factors in yeast. Methods Enzymol..

[43] Bagriantsev S.N., Kushnirov V.V., Liebman S.W. (2006). Analysis of amyloid aggregates using agarose gel electrophoresis. Methods Enzymol..

[44] Grishin A.V., Weiner J.L., Blumer K.J. (1994). Biochemical and genetic analysis of dominant-negative mutations affecting a yeast G-protein gamma subunit. Mol. Cell. Biol..

[45] Hirschman J.E., De Zutter G.S., Simonds W.F., Jenness D.D. (1997). The G beta gamma complex of the yeast pheromone response pathway. Subcellular fractionation and protein-protein interactions. J. Biol. Chem..

[46] Ahmed A.B., Znassi N., Chateau M.T., Kajava A.V. (2015). A structure-based approach to predict predisposition to amyloidosis. Alzheimers Dement..

[47] Halfmann R., Alberti S., Krishnan R., Lyle N., O’Donnell C.W., King O.D., Berger B., Pappu R.V., Lindquist S. (2011). Opposing effects of glutamine and asparagine govern prion formation by intrinsically disordered proteins. Mol. Cell.

[48] Chernova T.A., Kiktev D.A., Romanyuk A.V., Shanks J.R., Laur O., Ali M., Ghosh A., Kim D., Yang Z., Mang M. (2017). Yeast Short-Lived Actin-Associated Protein Forms a Metastable Prion in Response to Thermal Stress. Cell Rep..

[49] Toombs J.A., McCarty B.R., Ross E.D. (2010). Compositional determinants of prion formation in yeast. Mol. Cell. Biol..

[50] Derkatch I.L., Bradley M.E., Zhou P., Chernoff Y.O., Liebman S.W. (1997). Genetic and environmental factors affecting the de novo appearance of the [PSI+] prion in Saccharomyces cerevisiae. Genetics.

[51] Allen K.D., Chernova T.A., Tennant E.P., Wilkinson K.D., Chernoff Y.O. (2007). Effects of ubiquitin system alterations on the formation and loss of a yeast prion. J. Biol. Chem..

[52] Chernova T.A., Allen K.D., Wesoloski L.M., Shanks J.R., Chernoff Y.O., Wilkinson K.D. (2003). Pleiotropic effects of Ubp6 loss on drug sensitivities and yeast prion are due to depletion of the free ubiquitin pool. J. Biol. Chem..

[53] Chen P., Hochstrasser M. (1996). Autocatalytic subunit processing couples active site formation in the 20S proteasome to completion of assembly. Cell.

[54] Buchanan B.W., Lloyd M.E., Engle S.M., Rubenstein E.M. (2016). Cycloheximide Chase Analysis of Protein Degradation in *Saccharomyces cerevisiae*. J. Vis. Exp..

[55] Alberti S., Halfmann R., King O., Kapila A., Lindquist S. (2009). A systematic survey identifies prions and illuminates sequence features of prionogenic proteins. Cell.

[56] Bondarev S.A., Bondareva O.V., Zhouravleva G.A., Kajava A.V. (2018). BetaSerpentine: A bioinformatics tool for reconstruction of amyloid structures. Bioinformatics.

[57] Roche D.B., Villain E., Kajava A.V. (2017). Usage of a dataset of NMR resolved protein structures to test aggregation versus solubility prediction algorithms. Sci. A Publ. Protein Soc..

[58] Suzuki G., Tanaka M. (2013). Expanding the yeast prion world: Active prion conversion of non-glutamine/asparagine-rich Mod5 for cell survival. Prion.

[59] Maddelein M.L., Dos Reis S., Duvezin-Caubet S., Coulary-Salin B., Saupe S.J. (2002). Amyloid aggregates of the HET-s prion protein are infectious. Proc. Natl. Acad. Sci. USA.

[60] Sondheimer N., Lindquist S. (2000). Rnq1: An epigenetic modifier of protein function in yeast. Mol. Cell.

[61] Suzuki G., Shimazu N., Tanaka M. (2012). A yeast prion, Mod5, promotes acquired drug resistance and cell survival under environmental stress. Science.

[62] Schlissel G., Krzyzanowski M.K., Caudron F., Barral Y., Rine J. (2017). Aggregation of the Whi3 protein, not loss of heterochromatin, causes sterility in old yeast cells. Science.

[63] Sarnataro D. (2018). Attempt to Untangle the Prion-Like Misfolding Mechanism for Neurodegenerative Diseases. Int. J. Mol. Sci..

[64] Pepe A., Avolio R., Matassa D.S., Esposito F., Nitsch L., Zurzolo C., Paladino S., Sarnataro D. (2017). Regulation of sub-compartmental targeting and folding properties of the Prion-like protein Shadoo. Sci. Rep..

[65] Zhou P., Derkatch I.L., Liebman S.W. (2001). The relationship between visible intracellular aggregates that appear after overexpression of Sup35 and the yeast prion-like elements [PSI (+)] and [PIN *(+)]*. Mol. Microbiol..

[66] Mathur V., Taneja V., Sun Y., Liebman S.W. (2010). Analyzing the birth and propagation of two distinct prions, [PSI+] and [Het-s] (y), in yeast. Mol. Biol. Cell.

[67] Finegold A.A., Schafer W.R., Rine J., Whiteway M., Tamanoi F. (1990). Common modifications of trimeric G proteins and ras protein: Involvement of polyisoprenylation. Science.

[68] Ardito F., Giuliani M., Perrone D., Troiano G., Lo Muzio L. (2017). The crucial role of protein phosphorylation in cell signaling and its use as targeted therapy (Review). Int. J. Mol. Med..

[69] Liu Z., Li T., Li P., Wei N., Zhao Z., Liang H., Ji X., Chen W., Xue M., Wei J. (2015). The Ambiguous Relationship of Oxidative Stress, Tau Hyperphosphorylation, and Autophagy Dysfunction in Alzheimer’s Disease. Oxid. Med. Cell. Longev..

[70] Marcelli S., Corbo M., Iannuzzi F., Negri L., Blandini F., Nistico R., Feligioni M. (2018). The Involvement of Post-Translational Modifications in Alzheimer’s Disease. Curr. Alzheimer Res..

[71] Li H.Y., Yeh P.A., Chiu H.C., Tang C.Y., Tu B.P. (2011). Hyperphosphorylation as a defense mechanism to reduce TDP-43 aggregation. PLoS ONE.

[72] Nonaka T., Suzuki G., Tanaka Y., Kametani F., Hirai S., Okado H., Miyashita T., Saitoe M., Akiyama H., Masai H. (2016). Phosphorylation of TAR DNA-binding Protein of 43 kDa (TDP-43) by Truncated Casein Kinase 1delta Triggers Mislocalization and Accumulation of TDP-43. J. Biol. Chem..

[73] Rhoads S.N., Monahan Z.T., Yee D.S., Shewmaker F.P. (2018). The Role of Post-Translational Modifications on Prion-Like Aggregation and Liquid-Phase Separation of FUS. Int. J. Mol. Sci..

[74] Gnad F., de Godoy L.M., Cox J., Neuhauser N., Ren S., Olsen J.V., Mann M. (2009). High-accuracy identification and bioinformatic analysis of in vivo protein phosphorylation sites in yeast. Proteomics.

[75] Rangarajan N., Gordy C.L., Askew L., Bevill S.M., Elston T.C., Errede B., Hurst J.H., Kelley J.B., Sheetz J.B., Suzuki S.K. (2019). Systematic analysis of F-box proteins reveals a new branch of the yeast mating pathway. J. Biol. Chem..

[76] Caudron F., Barral Y. (2014). Mnemons: Encoding memory by protein super-assembly. Microb. Cell.

[77] Majumdar A., Cesario W.C., White-Grindley E., Jiang H., Ren F., Khan M.R., Li L., Choi E.M., Kannan K., Guo F. (2012). Critical role of amyloid-like oligomers of Drosophila Orb2 in the persistence of memory. Cell.

[78] Fioriti L., Myers C., Huang Y.Y., Li X., Stephan J.S., Trifilieff P., Colnaghi L., Kosmidis S., Drisaldi B., Pavlopoulos E. (2015). The Persistence of Hippocampal-Based Memory Requires Protein Synthesis Mediated by the Prion-like Protein CPEB3. Neuron.

[79] Gustin M.C., Albertyn J., Alexander M., Davenport K. (1998). MAP kinase pathways in the yeast *Saccharomyces cerevisiae*. Microbiol. Mol. Biol. Rev..

[80] Mendenhall M.D., Hodge A.E. (1998). Regulation of Cdc28 cyclin-dependent protein kinase activity during the cell cycle of the yeast *Saccharomyces cerevisiae*. Microbiol. Mol. Biol. Rev..

[81] Merlini L., Dudin O., Martin S.G. (2013). Mate and fuse: How yeast cells do it. Open Biol..

[82] Butty A.C., Pryciak P.M., Huang L.S., Herskowitz I., Peter M. (1998). The role of Far1p in linking the heterotrimeric G protein to polarity establishment proteins during yeast mating. Science.

[83] Nern A., Arkowitz R.A. (1999). A Cdc24p-Far1p-Gbetagamma protein complex required for yeast orientation during mating. J. Cell Biol..

[84] Nern A., Arkowitz R.A. (2000). G proteins mediate changes in cell shape by stabilizing the axis of polarity. Mol. Cell.

[85] Wang X., Tian W., Banh B.T., Statler B.M., Liang J., Stone D.E. (2019). Mating yeast cells use an intrinsic polarity site to assemble a pheromone-gradient tracking machine. J. Cell Biol..

[86] Madania A., Dumoulin P., Grava S., Kitamoto H., Scharer-Brodbeck C., Soulard A., Moreau V., Winsor B. (1999). The *Saccharomyces cerevisiae* homologue of human Wiskott-Aldrich syndrome protein Las17p interacts with the Arp2/3 complex. Mol. Biol. Cell.

[87] Chernova T.A., Chernoff Y.O., Wilkinson K.D. (2017). Prion-based memory of heat stress in yeast. Prion.

[88] Nagiec M.J., Dohlman H.G. (2012). Checkpoints in a yeast differentiation pathway coordinate signaling during hyperosmotic stress. PLoS Genet..

[89] Dupre D.J., Robitaille M., Rebois R.V., Hebert T.E. (2009). The role of Gbetagamma subunits in the organization, assembly, and function of GPCR signaling complexes. Annu. Rev. Pharmacol. Toxicol..

[90] Campanale J.P., Sun T.Y., Montell D.J. (2017). Development and dynamics of cell polarity at a glance. J. Cell Sci..

[91] Chen P., Johnson P., Sommer T., Jentsch S., Hochstrasser M. (1993). Multiple ubiquitin-conjugating enzymes participate in the in vivo degradation of the yeast MAT alpha 2 repressor. Cell.

[92] Longtine M.S., McKenzie A., Demarini D.J., Shah N.G., Wach A., Brachat A., Philippsen P., Pringle J.R. (1998). Additional modules for versatile and economical PCR-based gene deletion and modification in *Saccharomyces cerevisiae*. Yeast.

[93] Sikorski R.S., Hieter P. (1989). A system of shuttle vectors and yeast host strains designed for efficient manipulation of DNA in *Saccharomyces cerevisiae*. Genetics.

[94] Kiktev D.A., Patterson J.C., Muller S., Bariar B., Pan T., Chernoff Y.O. (2012). Regulation of chaperone effects on a yeast prion by cochaperone Sgt2. Mol. Cell. Biol..

[95] Sherman F. (2002). Getting started with yeast. Methods Enzymol..

[96] Dohlman H.G., Song J., Ma D., Courchesne W.E., Thorner J. (1996). Sst2, a negative regulator of pheromone signaling in the yeast *Saccharomyces cerevisiae*: Expression, localization, and genetic interaction and physical association with Gpa1 (the G-protein alpha subunit). Mol. Cell. Biol..

[97] Allen K.D., Wegrzyn R.D., Chernova T.A., Muller S., Newnam G.P., Winslett P.A., Wittich K.B., Wilkinson K.D., Chernoff Y.O. (2005). Hsp70 chaperones as modulators of prion life cycle: Novel effects of Ssa and Ssb on the Saccharomyces cerevisiae prion [PSI+]. Genetics.

[98] Kryndushkin D.S., Alexandrov I.M., Ter-Avanesyan M.D., Kushnirov V.V. (2003). Yeast [PSI+] prion aggregates are formed by small Sup35 polymers fragmented by Hsp104. J. Biol. Chem..

[99] Drozdova P.B., Barbitoff Y.A., Belousov M.V., Skitchenko R.K., Rogoza T.M., Leclercq J.Y., Kajava A.V., Matveenko A.G., Zhouravleva G.A., Bondarev S.A. (2020). Estimation of amyloid aggregate sizes with semi-denaturing detergent agarose gel electrophoresis and its limitations. Prion.

