# Supplementary material for: Aggregation and Prion-Inducing Properties of the G-Protein Gamma Subunit Ste18 are Regulated by Membrane Association
Source: Int J Mol Sci. 2020 Jul 16;21(14):5038. doi: 10.3390/ijms21145038 (PMC7403958; doi:10.3390/ijms21145038)
Supplement: Supplementary file 1 [file ijms-21-05038-s001.zip › Supplemental information 07.03.2020/2020.07.03. Supplemental Information.docx]

**Supplemental Information**

**Aggregation and Prion-Inducing Properties of the G-Protein Gamma Subunit Ste18 are Regulated by Association with the Plasma Membrane**

Tatiana A. Chernova, Zhen Yang, Tatiana A. Karpova^,^ John Shanks, Natalia Shcherbik, Keith D. Wilkinson and Yury O. Chernoff


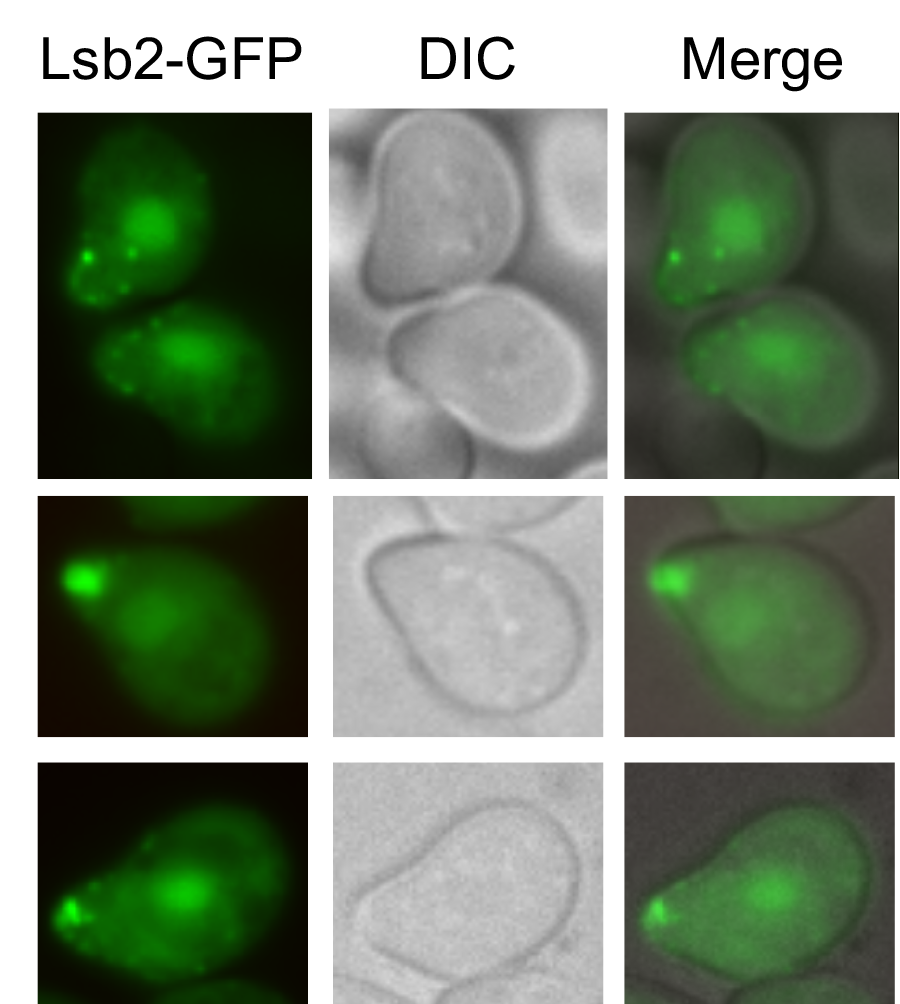


**Figure S1. Lsb2-GFP localizes in schmoo mating projection in** **the presence of pheromone.** Cells expressing Lsb2-GFP from *P_CUP1_* promoter were incubated with α-factor (10 µM) at 25°C for 1 hour. Live cells were visualized by fluorescence microscopy (GFP) and by differential interference microscopy (DIC) on the Olympus IX81 microscope.

**Movie S1. GFP-Ste18 co-localize with some de novo aggregate of Sup35NM-dsRED**

Cells simultaneously expressing GFP-Ste18 and Sup35-dsRED from *P_CUP1_* promoters were visualized by fluorescence microscopy on Delta Vision Imaging system.
